# Growth and Characterization of Graphene Layers on Different Kinds of Copper Surfaces

**DOI:** 10.3390/molecules27061789

**Published:** 2022-03-09

**Authors:** Peter M. Rafailov, Peter K. Sveshtarov, Vladimir B. Mehandzhiev, Ivalina Avramova, Penka Terziyska, Minko Petrov, Boyko Katranchev, Haritun Naradikian, Stefan I. Boyadjiev, Csaba Cserháti, Zoltán Erdélyi, Imre M. Szilágyi

**Affiliations:** 1 Institute of Solid State Physics, Bulgarian Academy of Sciences, 72 Tzarigradsko Chaussee Blvd., 1784 Sofia, Bulgaria; peter.sveshtarov@issp.bas.bg (P.K.S.); vmehandzhiev@issp.bas.bg (V.B.M.); penka@issp.bas.bg (P.T.); mpetrov@issp.bas.bg (M.P.); boykopk@issp.bas.bg (B.K.); harry@issp.bas.bg (H.N.); boiajiev@gmail.com (S.I.B.); 2 Institute of General and Inorganic Chemistry, Bulgarian Academy of Sciences, Acad. G. Bonchev Str., bl. 11, 1113 Sofia, Bulgaria; iva@svr.igic.bas.bg; 3 Department of Solid State Physics, Faculty of Sciences and Technology, University of Debrecen, P.O. Box 400, H-4002 Debrecen, Hungary; csaba.cserhati@science.unideb.hu (C.C.); zoltan.erdelyi@science.unideb.hu (Z.E.); 4 Department of Inorganic and Analytical Chemistry, Budapest University of Technology and Economics, Muegyetem rakpart 3, H-1111 Budapest, Hungary; szilagyi.imre.miklos@vbk.bme.hu

**Keywords:** graphene, chemical vapor deposition, Raman spectroscopy, X-ray photoelectron spectroscopy, ellipsometry, electron backscatter diffraction

## Abstract

Graphene films were grown by chemical vapor deposition on Cu foil. The obtained samples were characterized by Raman spectroscopy, ellipsometry, X-ray photoelectron spectroscopy and electron back-scatter diffraction. We discuss the time-dependent changes in the samples, estimate the thickness of emerging Cu_2_O beneath the graphene and check the orientation-dependent affinity to oxidation of distinct Cu grains, which also governs the manner in which the initial strong Cu-graphene coupling and strain in the graphene lattice is released. Effects of electropolishing on the quality and the Raman response of the grown graphene layers are studied by microtexture polarization analysis. The obtained data are compared with the Raman signal of graphene after transfer on glass substrate revealing the complex interaction of graphene with the Cu substrate.

## 1. Introduction

Since its discovery, graphene has been viewed as promising candidate for novel optoelectronic devices due to its extraordinary ballistic transport over micron lengths and large carrier mobility [1,2,3] combined with over 97% transparency for visible light [4]. In view of the growing interest in graphene, practical experience has established chemical vapor deposition (CVD) as the most prospective and readily accessible synthesis approach to satisfy the complex requirements of opto- and microelectronics for large-area and high-quality graphene with low density of structural defects [5]. Copper is used as a preferred substrate material due to its catalytic properties and because of the extremely low solubility of carbon in copper. Thus, procedures for the large-scale production of graphene were developed [6]. On the other hand, the CVD technology for graphene growth on metal catalysts has also revealed a potential for anticorrosive coating material in view of its chemical inertness and impermeability to most gases [7].

These optimistic prospects are, however, confronted by plenty of technological difficulties. The main problem is the challenge to achieve growth of nondefective graphene over sufficiently large areas. An important prerequisite for this goal is to have reliable backfeed by a standardized characterization of the obtained graphene directly on the metal substrate, which has to account for the whole complexity of graphene’s interactions with the metallic substrate/stabilizer, its possible oxides and gases/liquids which can come in contact with it during processing. As the commonly used CVD substrate is commercially obtained polycrystalline Cu foil, intensive research work was devoted to study the impact of Cu surface morphology and grain orientation on the grown graphene [8,9]. It has been found that Cu (111) surfaces grow the highest quality monolayer graphene while graphene grown on other low-index surfaces occasionally exhibits a multilayered structure and more defects [9]. The importance of Cu surface quality for the CVD growth is illustrated by the fact that some Cu surface defect patterns are reflected in corresponding defect patterns in the graphene layer even after its transfer into an isolating substrate [10]. An improvement of the Cu surface quality concerning smoothness and removal of chemical impurities can be achieved, e.g., by electropolishing [11].

Raman spectroscopy is a preferred characterization method for the study of graphene and the novel graphene-like 2D materials, as it is nondestructive, achieves high resolution with relatively simple commercially available setups and provides valuable information on the defect density, number of layers and their mechanical state. Most importantly, this technique reveals details of the electronic structure via the electron–phonon coupling and the rich single- and double-resonance phenomena characteristic for the Raman response of carbon-based materials. However, the versatility of this method is inherently connected to its main disadvantage—complex data interpretation—because of the need to account for the variability of many parameters. For instance, the combined plot of frequencies of the G-band (ω_G_) and 2D band (ω_2D_) of graphene is commonly used for quantifying strain and doping effects [12] but this approach yields correct results only to the first approximation, because strain and doping are not completely independent parameters, and the two bands are activated in the spectrum by two principally different resonant mechanisms [13]. Furthermore, it has been established that in freshly grown samples there is a strong van der Waals coupling of the graphene with the copper substrate [14]. This coupling not only imposes a strong compressive strain on the graphene layer due to the mismatch in the thermal expansion coefficients, but also diminishes its Raman intensity [15]. The scattering signal from the same graphene can be enhanced many times and the corresponding bands undergo redshift [16] through “decoupling” by means of, e.g., transferring the layer onto another substrate or by oxygen intercalation and oxidation processes on the copper surface [15,17]. It has also been reported that the rate of this oxidation and the facilitating or hindering role of graphene for this process depends on the surface crystallographic orientation of the Cu grains [17].

In this work, we study how Cu-graphene coupling is affected by oxygen penetration and copper surface oxidation on various time scales. For this purpose, we apply a combined approach including Raman and X-ray photoelectron spectroscopy (XPS), ellipsometry and electron back-scatter diffraction (EBSD). We find that immediately after CVD growth, the strong Cu-graphene coupling is still detectable in the XPS spectra in addition to its manifestation in the Raman spectra. In order to find a measure for the relaxation of this coupling with time, we use ellipsometry to estimate the thickness of the emerging Cu_2_O layer beneath the graphene. We also study the influence of the Cu surface orientation on its oxidation rate by means of EBSD orientation maps. Furthermore, the influence of Cu foil electropolishing on the quality of the grown graphene is studied by microtexture polarization analysis using a liquid crystal coating on the graphene layer. Although primarily applied for studying of liquid crystal properties, this method is very useful in the present case because the grain boundaries of the graphene domains become observable simultaneously with those of the copper grains.

## 2. Materials and Methods

Both raw (as supplied) and electropolished copper foil (99.8%, Alfa Aesar, 25 μm thick) was used as the growth substrate. Electropolishing was performed in 85% concentrated H_3_PO_4_ solution with the polished copper foil as the anode and a copper plate as the counter electrode at polarization voltage of 1.98–2.02 V at room temperature. Similar to [18], the anodic dissolution took place in mass-controlled kinetic regime, here for about 15 min, followed by thorough rinsing for 15 min in distilled/deionized water. The specimens sized 2 × 2 cm were then immediately transferred to the growth chamber. Electropolishing success was evaluated on control specimens by visual inspection for gloss and optical microscopy (×1000), comparing the surface to an untreated foil.

The graphene was grown in a cold wall Plasmalab System 100 research reactor of Oxford Instruments using low-pressure chemical vapor deposition (LPCVD). Low-pressure growth (4000 mTorr) was preferred because it yields lower graphene nucleation density [19] and the graphene growth-rate limiting process are the catalyst surface reactions (adsorption, 2D surface diffusion and desorption), in contrast to atmospheric pressure CVD, where the rate-limiting process is the precursor species’ diffusion towards the catalyst substrate [19,20] through the stagnant boundary layer. According to the established recipe for single-layer graphene growth, the CVD synthesis was performed in high-purity hydrogen (99.9999%) and methane (99.9995%) gas flow. Prior to this process, the Cu foil was annealed at 1065 °C for 30 min in argon/hydrogen atmosphere (1500 sccm/200 sccm, respectively). Subsequently, 10 sccm CH_4_ flow combined with 200 sccm H_2_ flow, were introduced for 30 min into the reaction chamber for the growth stage. Afterwards, the sample was quenched to 300 °C at 15 °C/min in hydrogen/argon atmosphere. Cooling to room temperature was done in high-purity argon atmosphere. The grown samples were kept in clean room environment prior to characterization and during aging.

Raman spectra were measured on a Jobin Yvon Labram HR-800 spectrometer with a CCD detector and entrance slits set to 1.5 cm^−1^ spectral width. The 632.8 nm line of a He/Ne-laser was applied for excitation using a 600 gr./mm grating, the absolute accuracy being about 1 cm^−1^. The laser beam was focused on a spot of about 1–2 μm in diameter on the sample surface using microscope optics with an objective of 50× magnification. The Raman measurements were performed on the graphene-coated Cu foils and after PMMA-assisted transfer onto glass substrates. The measured frequencies were calibrated by means of the T_2g_ vibration of silicon at 520.7 cm^−1^ and the lines corresponding to the stretching vibrations of oxygen and nitrogen at 1556 and 2331 cm^−1^, respectively, which are contained in the spectra.

The X-ray photoelectron spectroscopy (XPS) studies were performed in a VG Escalab MKII electron spectrometer using AlKα radiation with energy of 1486.6 eV under base pressure 10^−8^ Pa and a total instrumental resolution 1 eV. The binding energies (BE) were determined utilizing the C 1s line (from an adventitious carbon) as a reference with energy of 285.0 eV. The accuracy of the measured BE was 0.2 eV. The photoelectron lines of constituent element on the surface were recorded and corrected by subtracting a Shirley-type background and quantified using the peak area and Scofield’s photoionization cross-sections. The decovolution of spectra where is necessary was done with XPSPEAK41 software.

The ellipsometry measurements were performed using a Woollam M2000D rotating compensator spectroscopic ellipsometer with a wavelength range from 193 nm to 1000 nm in reflection mode. The involved data acquisition and analysis software was CompleteEASE 5.10 J. A. Woollam Co., Inc. (Lincoln, NE, USA) The spectroscopic ellipsometry data of Ψ and Δ were taken at room temperature at angles of incidence 50°, 60° and 70°.

The EBSD measurements were performed on a Thermo Fisher Scios 2 FIB/SEM instrument equipped with BRUKER e-FLASH FS EBSD detector with acceleration voltage 15 kV. Oxygen elemental distribution maps were measured by EDS (BRUKER XFlash^®^ 6–60) on specimens tilted at 70° with 5 kV acceleration voltage in order to find the appropriate surface. The high tilt angle and the lower acceleration voltage ensures the low penetration of the electrons, i.e., small interaction volume close to the specimen surface.

## 3. Results and Discussion

### 3.1. Sample Characterization with Raman and X-ray Photoelectron Spectroscopy

**Figure 1:**
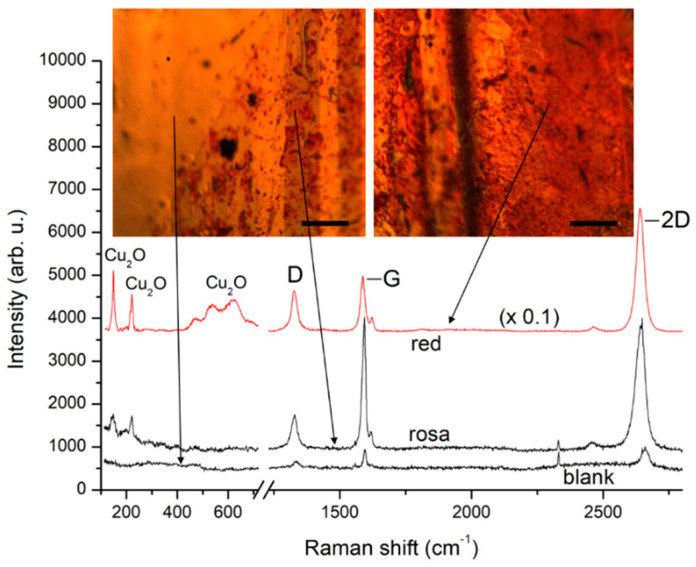
Micro-Raman spectra from different regions of a Gr/Cu foil several months after the CVD graphene growth process. The traces are plotted after background subtraction for clarity. Insets: microscopic images from the corresponding regions with exact specification of the spots of laser excitation. The scale bars represent 100 μm.

A typical case of time evolution of a graphene-coated Cu (Gr/Cu) foil is depicted in Figure 1. After prolonged storage at ambient conditions (typically longer than 6 months) the Gr/Cu foil exhibits regions with different color contrast under the optical microscope as seen from the two microscopic images in Figure 1. The difference in color contrast implies different oxidation degrees, with yellow color indicating blank copper, pale-red (or rosa) color indicating slight oxidation, and the redder tones indicating a more heavily oxidized state (Cu_2_O). When freshly taken out of the CVD chamber, the entire area of the foil looks similar to the left part of the left image with corresponding typical Raman spectrum as the “blank”-marked trace in Figure 1. The color changes visibly in the right part as well as in the whole right image are very nonuniform, indicating that some parts of the Gr/Cu surface are more resilient to oxidation than others. The Raman spectra also confirm the difference in the oxidation degrees with the “red”-marked spectrum displaying more than 10 times more intensive Cu_2_O bands at 149, 216, 525 and 640 cm^−1^ [21] than the “rosa”-marked one. On the other hand, the complete lack of such bands in the “blank”-marked spectrum and the clean yellow surface of the grain encompassing the left part of the left image in Figure 1 show that graphene has largely preserved this grain from oxidation. The Raman features of graphene in Figure 1 suggest that it is strongly coupled to Cu in the seemingly nonoxidized blank areas (low intensity and large blue shift of the G and 2D band [15]) while over the red areas this coupling is largely released and the corresponding Raman features change towards those of free-standing graphene.

To obtain a more quantitative picture of the oxidation development with time we performed a detailed characterization with different experimental methods on 3 samples with different storage times after CVD growth (6 months for the second and 20 months for the third sample) and with consecutive increasing density of reddish coloring. These samples will be referred to as “6 months” and “20 months” in what follows. The first sample referred to as “fresh” was examined immediately after graphene growth.

**Figure 2:**
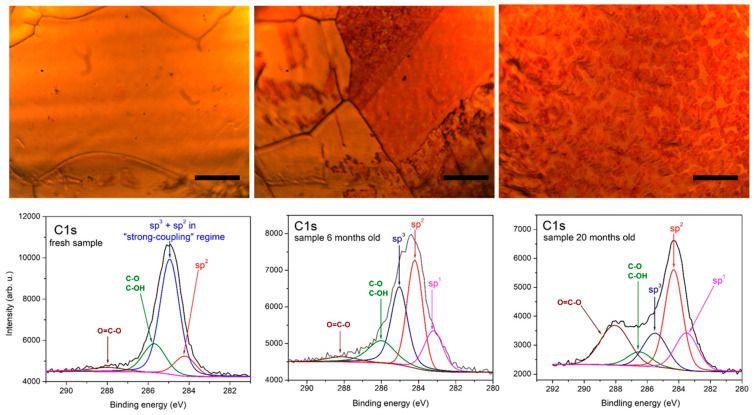
Microscopic images (**top**) of the three graphene-coated Cu foil samples (**left**—“fresh”; **middle**—”6 months”; **right**—“20 months”) with corresponding XPS spectra of the C1s region (**bottom**). The scale bars represent 50 μm. The C1s spectra are fitted according to the expected C-bond types. The fitting components are centered as follows: peak at 284.2 eV—red; peak at 284.9 eV—blue; peak at 285—285.5 eV—dark blue; peak around 286 eV—green; peak around 288 eV—brown, and peak around 283.5 eV—magenta.

**Figure 3:**
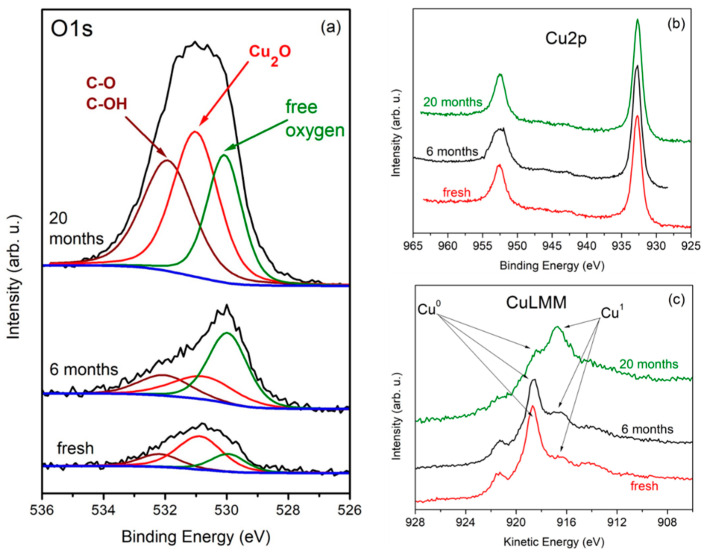
XPS spectra depicting the surface state of the Cu foil in the investigated three samples: (**a**) O1s spectra with fitting components—free O (green), O in Cu_2_O (red) O in C-O bonds (brown); (**b**) Cu 2p spectra; (**c**) Cu LMM Auger spectra.

A detailed XPS examination of the three samples was performed. Microscopic images and fitted C1s spectral bands are displayed in Figure 2. Figure 3 shows Cu LMM Auger spectra, Cu 2p spectra and O1s spectra for the three samples.

The C1s spectra are complex and their most intense component is typically the C1s peak of sp^2^ graphene at 284.2–284.4 eV. Interestingly, only for the “fresh” sample there is a peak at around 284.9 eV with much higher intensity than that of the conventional sp^2^ peak. This is consistent with the assumption that immediately after the growth process, the major part of the graphene layer is still strongly coupled to the copper substrate via exchange interactions between the Cu valence electrons and the carbon C1s core hole which yields a C1s sp^2^ signal at 284.75 eV [22]. In our case, this “strong-coupling” peak is slightly upshifted due to contributions from sp^3^ sites as well as defects in the graphene honeycomb lattice and C-H bonds whose signal is located between 285.0 and 285.5 eV. In the C1s spectra of the other samples these three contributions form a separate peak which is denoted as sp^3^ peak. Several other satellite peaks due to oxygen-containing groups are also detected. The peak at around 286 eV belongs to C-O/C-OH moieties, that around 288 eV to O=C-O bonds and carbonate groups, and that at ≈283 eV to sp^1^ type carbon bond in C_2_Cu. As expected, the oxygen-related peaks are more intense for longer storage time.

The O1s photoelectron spectra are also complex and were fitted with three peaks (see Figure 3a). According to the literature, the peak at the lower binding energy side at ~529.8 eV corresponds to an atomic phase state, i.e., a low-bound state [22,23,24,25] showing that there is a certain amount of free oxygen trapped by the graphene layer which serves as a reservoir for further Cu oxidation [26]. The middle peak at ~530.7 eV corresponds to Cu_2_O. As expected, this oxidation-related peak has maximum intensity for the sample with the longest storage time of 20 months. There is a shoulder at the higher binding energy side which is accounted for by the third peak at around 532 eV. The appearance of this shoulder is related to formation of C-O/C-OH and C=O bonds. From the Cu 2p photoelectron spectra in Figure 3b it is also obvious that the copper substrate is oxidized. The chemical states of copper were found to be Cu^0^ (Cu metal) and Cu^1+^ (Cu_2_O) for all studied samples. The same is evidenced by the Auger CuLMM line (see Figure 3c) where the oxidation-related peak gains intensity with increasing storage time.

**Figure 4:**
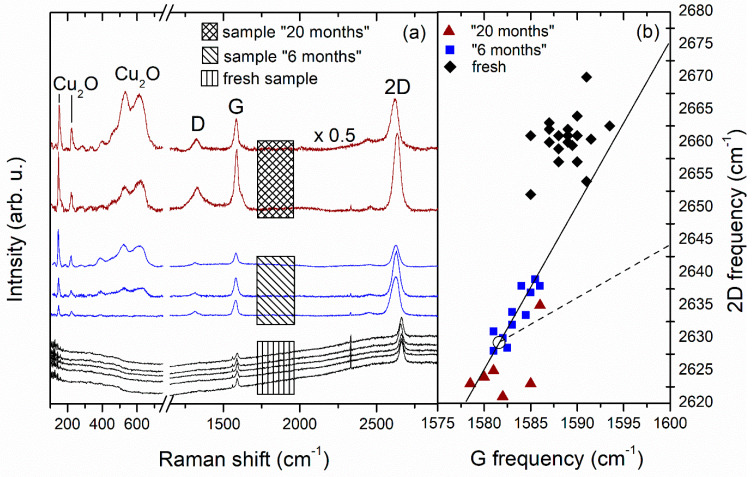
(**a**) Representative Raman spectra from the three investigated samples. Traces for samples “6 months” (blue) and “20 months” (brown) are plotted after background subtraction for clarity. Vibrational bands originating from graphene and Cu_2_O are indicated. (**b**) A 2D versus G frequency plot with data extracted from the spectra of the three investigated samples. Each point represents a (ω_G_, ω_2D_) pair from a distinct spectrum from sample: “fresh” (black diamonds), “6 months” (blue squares) and “20 months” (brown triangles). The straight solid line corresponds to a purely strain-induced shift, the empty circle to the zero-strain point and the dashed line to a purely doping-induced shift as taken from Figure 4 of Ref. [27].

Comprehensive spatially resolved Raman measurements were performed on the three samples. Representative spectra from this characterization are presented in Figure 4a. Recently it was shown for graphene grown on Cu that in ω_G_-ω_2D_ space the correlative shift of ω_G_ and ω_2D_ can be decomposed into a purely strain-induced shift [12] and a shift due to the fractional change in the effective Fermi velocity provided the graphene layers are doping-free [27]. As there is increasing evidence in the literature that the doping of graphene on Cu foil is negligible and thermally activated [28,29], we can apply a similar approach and show in Figure 4b the (ω_G_, ω_2D_) values from our measurements on the three samples. As can be appreciated from Figure 4a,b, the entire surface of the “fresh” sample displays a low-intensity graphene signal characteristic for strong coupling to the Cu substrate, with typical frequency values grouping around 1589 cm^−1^ for ω_G_ and around 2660 cm^−1^ for ω_2D_. This indicates significant compressive strain and, on the other hand, ω_2D_ is additionally upshifted due to the Fermi velocity reduction which is significant for our low excitation laser energy of 1.96 eV [27]. As the spectra of sample “6 months” were taken mostly from reddish-colored grains, there is a characteristic gathering of its (ω_G_, ω_2D_) values around the zero-strain point at (1581.6, 2629.3) cm^−1^ [27] with some points still indicating residual compressive strain. The Cu_2_O bands, which are completely missing in the “fresh”-sample spectra, display considerable intensity in the spectra of the two older samples. The (ω_G_, ω_2D_) values for sample “20 months” are more scattered with some points implying a transition to tensile strain imposed by local Cu_2_O aggregations [27]. It should, however, be pointed out that the strain release in the graphene lattice takes place in a very nonuniform way and there are still some isolated Cu grains in sample “6 months” which have preserved their initial “bare-copper” color and yield Raman spectra reminiscent of the strong-coupling regime.

From the collected Raman data, we obtained values for the peak intensity ratio of the 2D and G band I(2D)/ I(G) ranging from 2 to 2.8 which can, however, be impacted by the graphene-Cu coupling. In the Supporting Information we report a comprehensive Raman characterization of part of the “fresh” sample after transfer on glass substrate. As can be seen from Figure S1, I(2D)/ I(G) values obtained from this characterization range from 2.6 to 3.2 with a mean value of 2.9. The 2D bandwidth (FWHM) has a narrow distribution with mean value 30.8 cm^−1^ (compared to ~29 cm^−1^ before transfer). In Figure S2 we show scanning electron microscopy (SEM) images from the “fresh” sample and sample “6 months” displaying predominantly uniform brightness with occasional small regions of darker contrast. We interpret the combined Raman and SEM results as an indication for mainly single-layered graphene with possible minor presence of randomly stacked bilayer islands.

The oxygen content in the 3 examined samples as calculated from the XPS spectra is given in Table 1. Considering the XPS response of samples “6 months” and “20 months” shown in Figure 2 and Figure 3, it is noticeable that the increased presence of oxygen affects not only the Cu surface but also the graphene layer itself through various etching reactions. On the other hand, the D band intensity in the Raman spectra in Figure 1 and Figure 4 increases much stronger than that of the G and the 2D band in going from seemingly nonoxidized (blank) to highly oxidized (red) spots. This indicates that, with time, the defect density of the graphene coating of a certain copper grain becomes an important factor for formation and gradual enlargement of Cu_2_O patches on this grain. Still, we regard oxygen etching of graphene edges and grain boundaries as the main mechanism ensuring a slowly advancing oxidation of the substrate [9]. As was shown in [22], vacuum annealing at 700 °C can restore the strong graphene coupling to the Cu substrate. We expect that annealing in vacuum or inert atmosphere [30] at even higher temperatures approaching the CVD growth ones may help reducing the trap states in such “aged” graphene or recover its lattice ordering where it is locally deteriorated by oxygen-related etching reactions. The surface orientation of the Cu grain is another key factor for its oxidation rate and will be discussed in what follows.

### 3.2. Study of the Cu Surface Oxidation with Ellipsometry and Electron Backscattering Diffraction (EBSD)

To examine the weakening of the Cu-graphene coupling upon oxygen intercalation it is instructive to find a measure for the separation of the graphene layer from the Cu surface. We therefore estimated the thickness of the formed Cu_2_O layer by means of spectroscopic ellipsometry (SE) for the fresh and the 6-month-old sample. There are only few ellipsometric studies of as grown graphene on metals [31], while most of them are carried out on transferred graphene [32,33].

**Figure 5:**
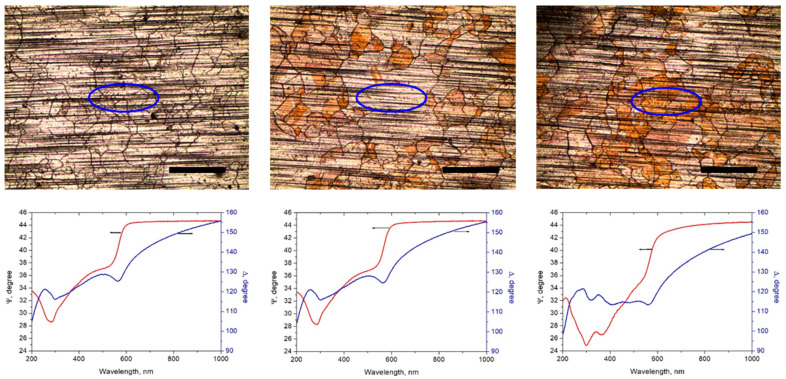
**Top**: Images of the SE-examined regions: “fresh” sample (**left**), low-oxidation region (**middle**) and high-oxidation region (**right**) of sample “6 months”. The scale bars represent 500 μm. **Bottom**: corresponding Ψ- and Δ–spectra acquired from the ellipse-shaped regions shown in the images (angle of incidence 60°).

As there is increasing evidence that the Cu oxidation rate beneath the graphene depends on the surface orientation of the copper grains [17], we examined two regions from sample “6 months”—one with predominant reddish color and one with apparently low oxidation degree. The regions from which the SE signal was collected, are shown in Figure 5 along with the corresponding dispersion spectra for the ellipsometric quantities Ψ and Δ.

The ellipsometric data for Ψ and Δ were modelled within a model consisting of two consecutive layers on top of an oblique substrate which is the copper foil. The first layer is Cu_2_O participating in the model with optical constants taken from Palik’s Handbook of Optical Constants [34] (CompleteEASE software). The second (top) layer is graphene with optical constants of graphite, taken again from [34]. The graphene layer thickness was assumed to be 0.35 nm as the combined Raman and SEM results indicate predominant monolayer graphene. Using this model, the following Cu_2_O average thicknesses were obtained: fresh sample: ≈0 nm; sample “6 months” low-oxidation region—0.5 nm; and sample “6 months” high-oxidation region—6.5 nm. From the strong variation of the oxide thickness and the obvious coincidence of the boundaries of differently colored regions with the Cu grain boundaries, as can be appreciated from the microphotographs in Figure 3 and Figure 5, it is obvious that the crystallographic orientation of the Cu surface has a key influence on the rate and degree of its oxidation.

To check this influence in detail, we examined the “fresh” sample and “6 months” sample by the EBSD technique. This study was conducted 2 months later in order to have initial Cu oxidation in the “fresh” sample. As this method has an effective probing depth above 10 nm, it can be safely assumed that the graphene and the oxide layer do not mimic the EBSD signal, which is almost entirely collected from the underlying copper [17]. Cu surface orientation maps were obtained by EBSD and, simultaneously, oxygen elemental distribution maps were measured by energy-dispersive X-ray spectroscopy (EDX) on the same spots in order to correlate the Cu orientation to the Cu_2_O content. The results were presented using the MTEX Matlab toolbox [35].

**Figure 6:**
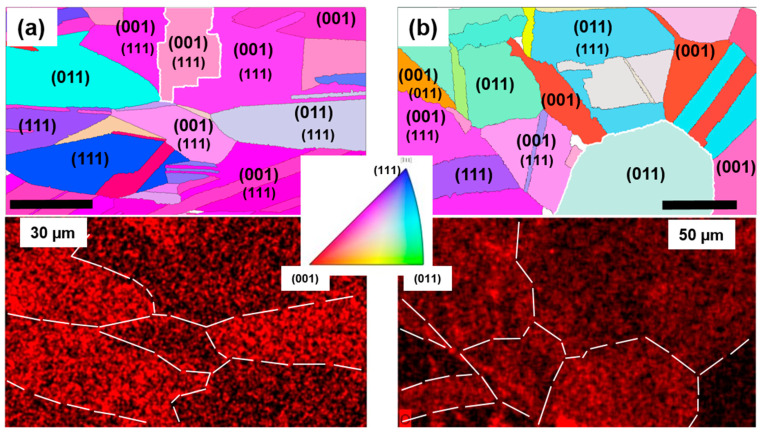
(**a**) EBSD orientation map of the investigated spot on the “fresh” sample (**top**) with corresponding EDX oxygen elemental distribution map (**bottom**). (**b**) Same as (**a**) for sample “6 months”. In the **top** panels, for grains with incomplete low-index orientation, the minor component is indicated in smaller font below the dominant one. **Bottom** panels: some Cu grains are delineated by a dashed line for clarity; the intensity of the red coloring corresponds to the oxygen concentration. Inset: orientation color code.

Figure 6 shows EBSD and EDX results for the “fresh” sample and “6 months” sample. As can be expected for a polycrystalline Cu foil, the majority of the grains are not perfectly oriented according to the basic Miller indices of the m3m structure. However, from the depicted orientation maps, it is noticeable that in both graphene-coated samples, predominantly (011) oriented Cu grains are most susceptible to Cu_2_O formation while grains with dominant (001) orientation exhibit the lowest oxidation rate. Grains with orientation near to (111) display an intermediate affinity towards oxidation. Surprisingly, these results are the opposite to those reported in [17], where for graphene-coated Cu foils the (001) grains were found to be most susceptible to oxidation and (011) grains most resilient to it. A possible explanation for this contradiction may lie in different substrate pretreatment and different details in the growth recipe. Our foil substrates were electropolished while those used in [11] were annealed in air at 250 °C prior to the CVD growth process. Under the preparation conditions applied in this study, rippling of the Cu surface and formation of terrace step bunches, while occasionally present, could be avoided to a large extent. As was shown by Hu et al. [36], the parameters of the initial stages of the CVD prior to the growth stage itself can significantly influence the physical properties of the Cu substrate. Moreover, a heat-treatment experiment performed by Wood et al. [9] on polycrystalline Cu foils with graphene revealed that (011) grains are the first among low-index grains to succumb to oxidation when heated. In Figure S3 of the Supporting Information we present EBSD results on two other Gr/Cu foil samples which confirm the observed dependence of the oxidation rate on the Cu grain surface orientation.

### 3.3. Examination of the Effects of Electropolishing

**Figure 7:**
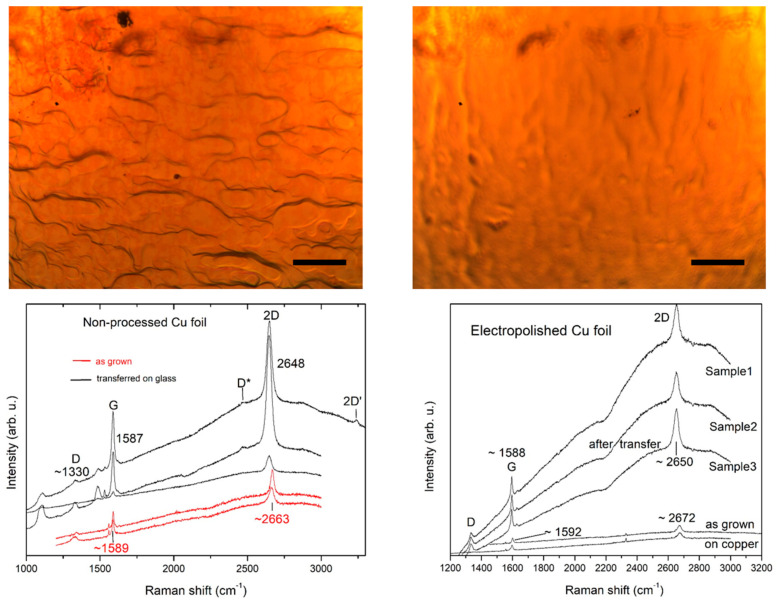
**Top** panel: Microscopic images of a nonprocessed (**left**) and an electropolished (**right**) Cu foil with graphene taken 1 week after the CVD growth. The scale bars represent 50 μm. **Bottom** panel: corresponding Raman spectra from the graphene coatings on the Cu foils shown in the top panel taken before and after transfer on glass substrates.

Electropolishing has been frequently applied as part of the Cu foil pretreatment to lower its surface roughness and to achieve better quality of the grown graphene [37,38]. Here, we compare the characteristics of graphene grown on Cu foil electropolished prior to the CVD growth and on Cu foil that has not undergone such pretreatment. The results from monitoring with optical microscopy and Raman spectroscopy are depicted in Figure 7. It is seen from a comparison of the microscopic images that the electropolished foil exhibits a better resistance to Cu oxidation, while the unprocessed Gr/Cu foil shows precursors of pale-red coloring already 1 week after the graphene growth process.

From the Raman spectra in Figure 7, it is seen that graphene grown on electropolished Cu foil experiences a stronger coupling to the Cu substrate and a stronger strain (2D band redshift of more than 20 cm^−1^ after transfer onto glass as compared to ~15 cm^−1^ for unprocessed Cu foil). It is also found that the Cu-graphene coupling makes the intensity ratio of the 2D and G band less informative for determination of the number of layers and it becomes a reliable parameter only after the transfer on isolating substrate.

**Figure 8:**
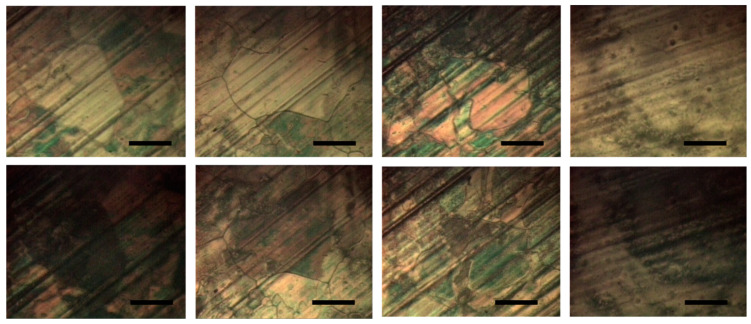
Four vertical pairs of microphotographs of E7-coated graphene (**top**—bright state; **bottom**—dark state achieved after rotation at 45°). The images depict: graphene grains on electropolished Cu foil (**left** two pairs) and on nonprocessed Cu foil (**right** two pairs). The scale bars represent 100 μm.

To further illustrate the effect of electropolishing, we visualize the domains in the graphene layers grown on an electropolished and on a nonprocessed Cu foil by means of the nematic liquid crystal (LC) E7. This is possible due to the interaction of two-dimensional graphene-honeycomb structure with the LC benzene rings through π–π electron stacking (binding energy of −2eV) which induces a planar alignment of the LC, thus creating LC pseudonematic domains (PNDs) at the graphene surface [39]. As the graphene layer consists of grains with different crystallographic orientation and each grain imposes its own ordering on the LC, the shapes and boundaries of these anisotropic LC domains strongly correlate with those of the graphene grains. When observed through a reflected cross-polarized microscope, such PNDs with homogeneous planar alignment transit from a bright to a dark state upon rotation at 45° [39]. Distinct graphene grains can thus be visualized [39] and observed simultaneously with the copper grains. Figure 8 shows pairs of such microphotographs taken from E7-coated graphene grown on electropolished and nonprocessed Cu foil. As is seen, the PNDs formed on electropolished Cu foil (Figure 8, left two pairs) are large and well outlined with clear boundary walls suggesting that the LC has achieved a uniform planar orientation. This indicates a high crystalline quality of the underlying graphene which is an important prerequisite for the above described π–π stacking mechanism. Thus, larger graphene grains are found on electropolished Cu foil which can overgrow Cu grain boundaries presumably due to the smaller Cu surface roughness. On the other hand, the PNDs in the right two image pairs in Figure 8 (nonprocessed Cu foil) display enhanced birefringence (recognized by the red coloring) and unclear boundaries which are signs for nonuniform and deteriorated planar alignment. We attribute this to the smaller graphene grains with higher defect density on the nonprocessed Cu foil, which cannot achieve an efficient π–π stacking interaction with the LC. Indeed, the Raman spectra of graphene on electropolished foil in Figure 7 completely lack a D band compared to the well-discernible D bands in the spectra from the nonprocessed foil.

## 4. Conclusions

We investigated single-layer graphene-coated Cu foils with respect to the Cu-graphene coupling and Cu oxidation to Cu_2_O as a way for its relaxation. Correlating results were obtained from Raman and XPS characterization, ellipsometry and the EBSD technique. We find strong Cu-graphene coupling on the nonoxidized Cu grains and gradual release of this coupling along the “strain” line without significant doping effects. Our ellipsometric results provide an estimate for the typical oxide layer thickness beneath the graphene after 6 months of sample aging, which varies from fractional parts of a nanometer to 6–7 nm. We confirm that in the case of polycrystalline foil oxidation to Cu_2_O of graphene-coated copper is grain-selective, with (001)-oriented grains being most resilient and (011)-oriented ones most susceptible to oxidation. Correspondingly, the strong Cu-graphene coupling and the strain in the graphene lattice is released very nonuniformly and over different time scales.

From a visualization of graphene grains by means of coating by liquid crystal, it is found that graphene grown on electropolished Cu foil exhibits larger grains with lower defect density. It is found that the Cu-graphene coupling alters the frequency and the intensity ratio of the G and 2D band, and a determination of the number of graphene layers from Raman spectral parameters should preferably be made after transfer on isolating substrate.

## Figures and Tables

**Table 1:** Oxygen content as determined from the XPS data.

Sample	Time Passed after CVD Growth	O, at%
“fresh”	1–2 days	9.54
“6 months”	6 months	18.32
“20 months”	20 months	33.80

## Data Availability

The data presented in this study are available on request from the authors.

## Supplementary Materials

The following supporting information can be downloaded at: https://www.mdpi.com/article/10.3390/molecules27061789/s1. Figure S1: Raman characterization of transferred graphene; Figure S2: SEM characterization; Figure S3: EBSD results from other samples and corresponding discussion.
