# Supplementary material for: Growth and Characterization of Graphene Layers on Different Kinds of Copper Surfaces
Source: Molecules. 2022 Mar 9;27(6):1789. doi: 10.3390/molecules27061789 (PMC8956068; doi:10.3390/molecules27061789)
Supplement: Supplementary file 1 [file molecules-27-01789-s001.zip › molecules-1562310-supplementary.pdf]

molecules

Supplementary Materials

Growth and Characterization of Graphene Layers on Different Kinds of Copper Surfaces

Peter M. Rafailov [1,*], Peter K. Sveshtarov [1], Vladimir B. Mehandzhiev [1], Ivalina Avramova [2], Penka Terziyska [1], Minko Petrov [1], Boyko Katranchev [1], Haritun Naradikian [1], Stefan I. Boyadjiev [1], Csaba Cserháti [3], Zoltán Erdélyi [3] and Imre M. Szilágyi [4]

1 Institute of Solid State Physics, Bulgarian Academy of Sciences, 72 Tzarigradsko Chaussee Blvd., 1784 Sofia, Bulgaria; rafailov@issp.bas.bg (P.R.)
2 Institute of General and Inorganic Chemistry, Bulgarian Academy of Sciences, Acad. G. Bonchev Str., bl. 11, 1113 Sofia, Bulgaria; iva@svr.igic.bas.bg (I.A.)
3 Department of Solid State Physics, Faculty of Sciences and Technology, University of Debrecen, P. O. Box 400, H-4002 Debrecen, Hungary; zoltan.erdelyi@science.unideb.hu (Z.E.)
4 Department of Inorganic and Analytical Chemistry, Budapest University of Technology and Economics, Muegyetem rakpart 3, H-1111 Budapest, Hungary; szilagyi.imre.miklos@vbk.bme.hu (I.S.)
* Correspondence: rafailov@issp.bas.bg;

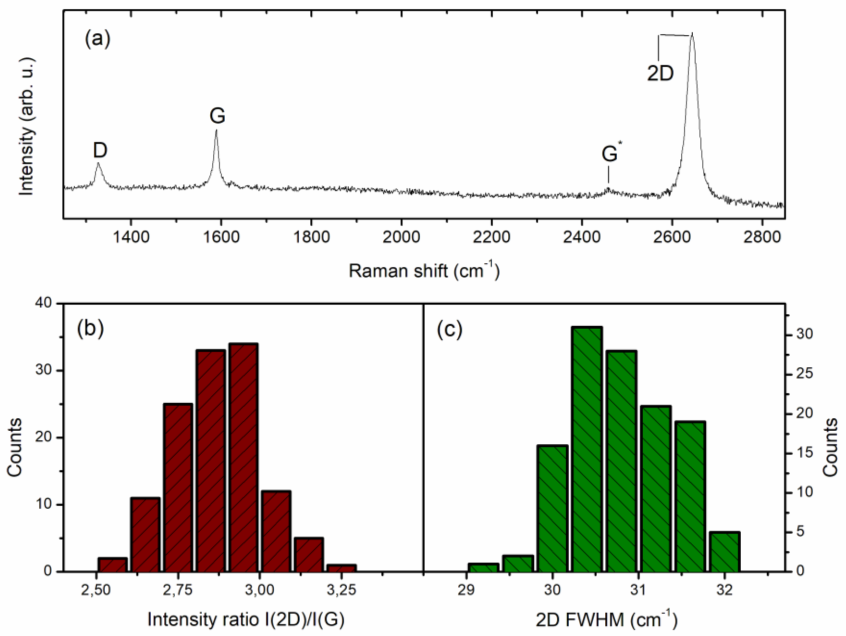

Figure S1. (**a**) Representative Raman spectrum from comprehensive spatially resolved Raman characterization of the “fresh” sample after transfer onto glass substrate. The main spectral bands 2D and G, the defect-related D band as well as the characteristic combination band G* are denoted. (**b**) Histogram of the peak intensity ratio I(2D)/I(G) values for transferred graphene. (**c**) Same as (**b**) for the 2D bandwidth.

The first Raman characterization of the “fresh” sample (for sample description see main article) was done in an as-grown state on Cu. From those data we obtained values for the peak intensity ratio of the 2D and G band I(2D)/I(G) ranging from 2 to 2.8. However, this ratio has limited informative value for graphene on Cu because the layer/substrate coupling may impact 2D and G band in different manner. Therefore, we transferred a part of this sample on glass substrate and performed comprehensive spatially resolved Raman measurements. The obtained results are depicted in Fig. S1. We found the G mode

at ~1585 and the 2D band at ~2640 cm^{-1}. Although the D band intensity in the spectra measured on Cu is negligible (see Figure 4a in the mail article), after transfer there is already a well-formed D band which is attributed to defects introduced by the transfer process. I(2D)/I(G) ranges from 2.6 to 3.2 with a mean value of 2.9. The 2D bandwidth (FWHM) has a narrow distribution with mean value 30.8 cm^{-1} (compared to ~29 cm^{-1} before transfer). These values speak for the predominant monolayer composition [1] of the graphene in the "fresh" sample; however, they cannot rule out minor presence of bilayer and multilayer islands [2].

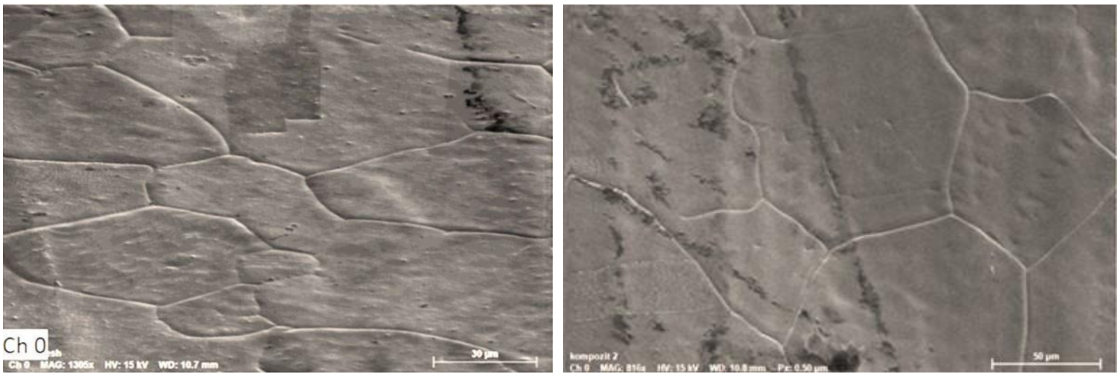

Figure S2. SEM images corresponding to the EBSD-investigated spots shown in Figure 6 of the "fresh" sample (left) and sample "6 months" (right).

We also conducted a SEM characterization of the "fresh" sample and "6 months" sample on the same spots on which the EBSD measurements were done (see Figure 6 in the main article). The SEM images are shown in Figure S2. Besides the information about the surface morphology of the EBSD-investigated spots, these images exhibit a relatively uniform contrast with relatively small presence of distinctively darker areas which could indicate an additional graphene layer. Such an additional layer should manifest itself also in stronger blocking of secondary electrons from the substrate, thus leading to lower brightness in the images [3,4]. Such a case is found in the middle of the upper part of Fig. S2 (left): a square-like spot with markedly darker contrast which corresponds to a (001) oriented grain in Fig. 6a (main article). SEM imaging itself is insufficient to unambiguously identify a bilayer island, but inspection of both panels of Fig. 6a reveals that the surrounding area also has a dominant (001) orientation which is known to be more likely to host multilayer graphene [5] and this local region exhibits a uniform low Cu_2O content which does not correlate with the contrast. We therefore attribute the darker contrast on this particular grain to a bilayer island. The uniform brightness in the most part of the SEM images combined with the Raman results on ratio and FWHM (2D) thus indicate that the graphene in the examined samples is predominantly single-layered with possible minor presence of twisted bilayer graphene.

To test the found dependence of the oxidation degree on the orientation of the copper grains, we performed the same EBSD measurements on a control pair of samples with the same graphene growth conditions and similar aging history: a polycrystalline Cu foil stored for 2 months after graphene growth (referred to as C1) and a foil with dominant (111) Cu surface orientation with occasional presence of (001) grains 10 months after graphene growth (sample C2). The results are depicted in Figure S3. For the textured sample it is seen that the (001) grains are less oxidized than the predominantly (111) oriented surface. (011)-oriented Cu grains in sample C2 are most susceptible to Cu_2O formation. These results are in agreement with those obtained for the "fresh" sample and "6 months" sample (see Figure 6 in the main article), thus confirming the reproducibility of the established correlation.

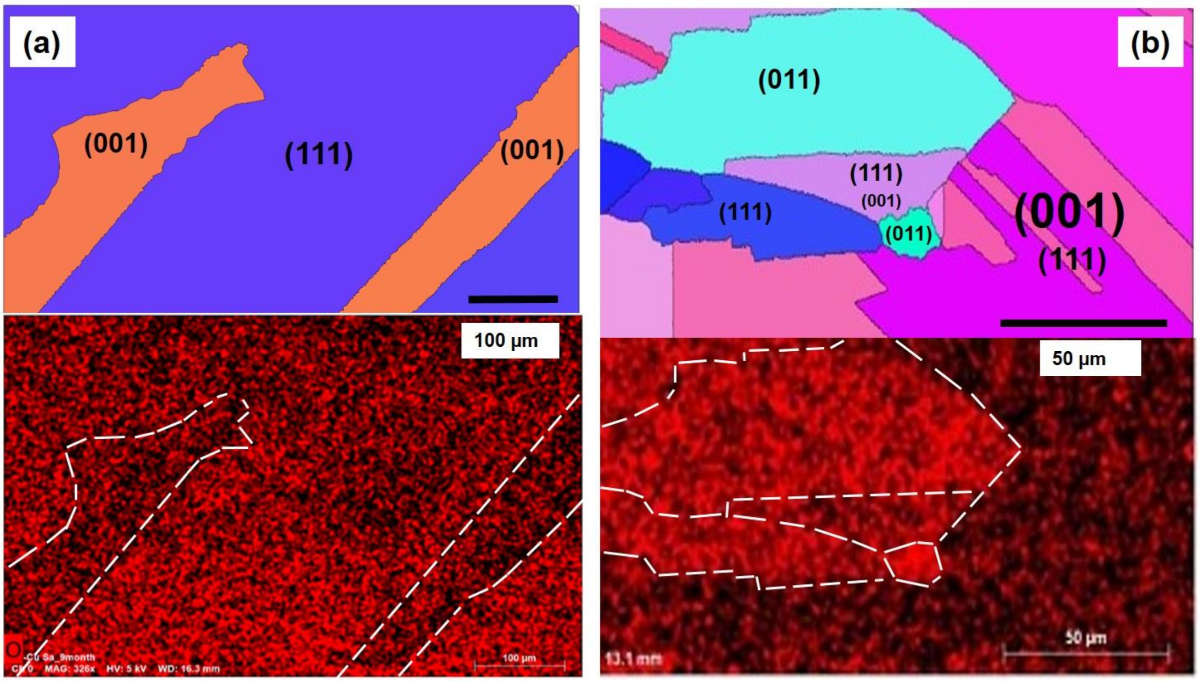

Figure S3. (**a**) EBSD orientation map of the investigated spot in sample C2 (top) with corresponding oxygen elemental distribution map (bottom). (**b**) Same as (**a**) for sample C1. In the top panels, for grains with incomplete low-index orientation, the minor component is given in smaller font below the dominant one. Bottom panels: some Cu grains are delineated by a dashed line for clarity; the intensity of the red coloring corresponds to the oxygen concentration.

References

1. Ferrari, A. C.; Meyer, J. C.; Scardaci, V.; Casiraghi, C.; Lazzeri, M.; Mauri, F.; Piscanec, S.; Jiang, D.; Novoselov, K. S.; Roth, S.; Geim, A. K. Phys. Rev. Lett. **2006**, *97*, 187401.
2. Bae, S.; Kim, H.; Lee, Y.; Xu, X.; Park, J.-S.; Zheng, Y.; Balakrishnan, J.; Lei, T.; Ri Kim, H.; Song, Y.I.; Kim, Y.-J.; Kim, K.S.; Özyilmaz, B.; Ahn, J.-H.; Hong, B.H.; Iijima, S. Roll-to-roll production of 30-inch graphene films for transparent electrodes. Nature Nanotechnology **2010**, *5*, 574-578.
3. Lee, J.; Zheng, X.; Roberts, R.C.; Feng, P.X.-L. Scanning electron microscopy characterization of structural features in suspended and non-suspended graphene by customized CVD growth, Diamond Relat.Mater. 54 (2015) 64.
4. Erdman, N.; Bell, D.C.; Reichelt, R. Scanning Electron Microscopy. In Springer Handbook of Microscopy; Hawkes, P., Spence, J.C., Eds.; Springer International Publishing: Cham, Switzerland, 2019; pp. 229–318.
5. Wood, J.D.; Schmucker, S.W.; Lyons, A.S.; Pop, E.; Lyding, J.W. Effects of polycrystalline Cu substrate on graphene growth by chemical vapor deposition, Supporting Information, Nano Lett. 11 (2011) 4547.
